# Cause-Specific Mortality According to Urine Albumin Creatinine Ratio in the General Population

**DOI:** 10.1371/journal.pone.0093212

**Published:** 2014-03-27

**Authors:** Tea Skaaby, Lise Lotte Nystrup Husemoen, Tarunveer Singh Ahluwalia, Peter Rossing, Torben Jørgensen, Betina Heinsbæk Thuesen, Charlotta Pisinger, Knud Rasmussen, Allan Linneberg

**Affiliations:** 1 Research Centre for Prevention and Health, Glostrup University Hospital, Glostrup, Denmark; 2 Section of Metabolic Genetics, University of Copenhagen, Copenhagen, Denmark; 3 Steno Diabetes Center, Gentofte, Denmark; 4 University of Copenhagen, Copenhagen, Denmark; 5 University of Aarhus, Aarhus, Denmark; 6 Faculty of Health Science, University of Copenhagen, Copenhagen, Denmark; 7 Faculty of Medicine, Alborg University, Alborg, Denmark; 8 Department of Medicine, Roskilde University Hospital, Roskilde, Denmark; 9 Department of Clinical Experimental Research, Glostrup University Hospital, Glostrup, Denmark; 10 Department of Clinical Medicine, Faculty of Health and Medical Sciences, University of Copenhagen, Copenhagen, Denmark; Kagoshima University Graduate School of Medical and Dental Sciences, Japan

## Abstract

**Background:**

Urine albumin creatinine ratio, UACR, is positively associated with all-cause mortality, cardiovascular disease and diabetes in observational studies. Whether a high UACR is also associated with other causes of death is unclear. We investigated the association between UACR and cause-specific mortality.

**Methods:**

We included a total of 9,125 individuals from two population-based studies, Monica10 and Inter99, conducted in 1993–94 and 1999–2001, respectively. Urine albumin creatinine ratio was measured from spot urine samples by standard methods. Information on causes of death was obtained from The Danish Register of Causes of Death until 31 December 2010. There were a total of 920 deaths, and the median follow-up was 11.3 years.

**Results:**

Multivariable Cox regression analyses with age as underlying time axis showed statistically significant positive associations between UACR status and risk of all-cause mortality, endocrine nutritional and metabolic diseases, mental and behavioural disorders, diseases of the circulatory system, and diseases of the respiratory system with hazard ratios 1.56, 6.98, 2.34, 2.03, and 1.91, for the fourth UACR compared with the first, respectively. Using UACR as a continuous variable, we also found a statistically significant positive association with risk of death caused by diseases of the digestive system with a hazard ratio of 1.02 per 10 mg/g higher UACR.

**Conclusion:**

We found statistically significant positive associations between baseline UACR and death from all-cause mortality, endocrine nutritional and metabolic diseases, and diseases of the circulatory system and possibly mental and behavioural disorders, and diseases of the respiratory and digestive system.

## Introduction

Chronic kidney disease (CKD) affects 10–16% of adults worldwide [1], [2]. The 2002 Kidney Disease Outcomes Quality Initiative guidelines define chronic kidney disease as persistent kidney damage, usually characterized by albuminuria or reduced glomerular filtration rate (GFR) [3]. Obese, individuals with diabetes and individuals with the metabolic syndrome often have albuminuria. Also, children born small for gestational age have an increased risk of developing renal impairment –or hypertension—later in life possibly due to renal programming or subclinical disease.

Microalbuminuria occurs when the glomerular albumin permeability is abnormally high, and the kidneys leak small amounts of albumin into the urine. It is a marker of vascular damage and endothelial dysfunction and an early marker of chronic kidney disease. The pathophysiology of the development is not fully understood but most likely, it involves a change in the intrarenal hemodynamics. An accepted theory is that above-normal urine albumin creatinine (UACR) is both a cause and a consequence of vascular damage [4].

Microalbuminuria can be diagnosed from the urine excretion of albumin in a 24-hour urine collection or, more frequently, from the albumin creatinine ratio in a spot sample. The most commonly used classification defines a level of 10 mg/g and 15 mg/g as the upper normal limit for men and women, respectively [4]. Higher levels are classified as high normal, micro- and macroalbuminuria [4].

Albuminuria is associated with an increased risk of renal function loss, cardiovascular and end stage renal disease, cardiovascular and all-cause mortality [3]–[8]. Albuminuria independently predicted cardiovascular events among normoalbuminuric patients with type 2 diabetes in a study by Ruggenenti et al [9]. It is an independent predictor of stroke, possibly even better than other markers of renal function such as glomerular filtration rate (GFR) and cystatin C [10]. Besides, albuminuria is in observational studies associated with other disorders such as cognitive impairment and chronic obstructive pulmonary disease (COPD) [11], [12]. Since a decrease in urine albumin excretion is associated with a lower risk of cardiovascular and renal disease [13], [14], microalbuminuria is an important therapeutic target.

We aimed to investigate whether other causes of death beside cardiovascular disease and diabetes contribute to the association between UACR and risk of dying. We investigated the prospective association between UACR and cause-specific mortality according to The International Classification of Disease (ICD) in two cohorts from a general Danish population.

## Methods

### Ethics Statement

Participants gave their written informed consent, and the studies were approved by the local ethics committee of Copenhagen County and the Danish Data Protection Agency. The recommendations of the Declaration of Helsinki were followed.

### Study populations

We used the two population based studies, Monica10 and Inter99. The Monica10 study carried out in 1993–1994 was a 10 year follow-up study of the MonicaI study conducted in 1982–1984. The MonicaI population was recruited from the Danish Central Personal Register as an age- and sex-stratified random sample of the population. The Monica10 study included 2,656 individuals between the ages 40–71 years, and the participation rate was 64.3% [15]. A total of 2,654 participants from the Monica10 study with measurements of UACR were included in the study.

The Inter99 study that was carried out in 1999–2001 included 13,016 individuals aged 30–60 years drawn from an age- and sex-stratified random sample of the population in the same area as MonicaI [16]. A total of 6,784 persons participated yielding a baseline participation rate of 52.5%. The Inter99 study was a population-based randomized controlled trial (CT00289237, ClinicalTrials.gov) set to investigate the effects of lifestyle intervention on cardiovascular disease (CVD) [16]. A total of 6,471 participants from Inter99 with measurements of UACR were included in the present study. Inter99 data were considered observational, and analyses were adjusted for study group.

Both studies included a self-administered questionnaire, a physical examination, various blood tests (fasting), and a random urinary spot sample. The questionnaires provided information on education, leisure time physical activity, smoking habits, alcohol consumption and a self-reported diagnosis of diabetes. Height and weight were measured without shoes and with light clothes. Body mass index (BMI) was calculated as weight (kg) divided by height (m) squared. Blood pressure (mmHg) was measured twice in the sitting position, and the average of the two systolic measurements was used.

### Exposure

In the Monica10 study, the urine albumin concentration was determined by a standard turbidimetric method (Hitachi 717 analyzer; Roche Diagnostics) on a single morning urine specimen [17]. The urine albumine measurement range was 1.52–3700 mg/l (intra-assay coefficient of variation, CV = 2.2%, inter-assay CV = 9.4%). Urine creatinine was analyzed with the Jaffe' reaction without deproteinizing and then quantified by a photometric method (Hitachi 717 analyzer; Roche Diagnostics). UACR was calculated.

In the Inter99 study, the urine samples were cooled and analyzed within a week from sampling. An internal validation study with a urine albumin measuring range of 2.2–4420 mg/l confirmed the durability of urine albumin in urine samples kept at 2–8°C. Urine albumin concentration was analysed by a turbidimetric assay (Cobas Mira plus, Roche diagnostic systems, la Roche, Basel, Switzerland). The urine albumine measurement range was 2.2–8000 mg/l (intra-assay CV = 2.1%, inter-assay CV = 8.3%). Internal validation ensured that the results were comparable throughout the study period. Urine creatinine concentration was analysed by the Jaffé method (Hitachi 912 system, Roche diagnostic, Germany).

### Other covariates

Participants were classified in the following way (“study group”): no intervention [participants from Monica10], lifestyle counseling [group B from Inter99], lifestyle and group counseling [group A from Inter99]). Education was classified as no education beyond basic or education including students. Physical activity during leisure time was divided into sedentary, light, or moderate/vigorous. Smoking habits were divided into never smokers, ex-smokers, occasional smokers, current smokers <15 g/day including occasional smokers, 15-<25 g/day, or ≥25 g of tobacco/day. BMI was divided into the following groups: <18.5, ≥18.5–25, ≥25–30, or ≥30 kg/m^2^. Alcohol consumption was classified as consumption of 0, >0–7, >7–14, or >14 standard drinks per week.

The lipid profile was determined by enzymatic colorimetric methods (Roche, Mannheim, Germany) [16], [18]–[20]. Fasting plasma glucose was measured by the hexokinase/glucose-6-phosphate dehydrogenase assay (Roche Diagnostics, former Boehringer Mannheim, Germany) [16], [19], [21].

### Registry-based diagnoses

All residents in Denmark have a unique and permanent personal civil registration number allowing linkage of data from complete national registers on an individual level. The Danish Registry of Causes of Death provided up to three diagnoses suspected to be the cause of death (we used only the first one) [22]. The major groups of diagnoses of death were (all ICD-10): neoplasms, C00–D48; endocrine, nutritional and metabolic diseases, E00–E90; mental and behavioural disorders, F00–F99; diseases of the nervous system, G00–G99; diseases of the circulatory system, I00–I99; diseases of the respiratory system, J00–J99; and diseases of the digestive system, K00–K93. The remaining deaths (n = 109) consisted mainly of deaths caused by “Symptoms, signs and abnormal clinical and laboratory findings, not elsewhere classified” (R) and external causes of morbidity and mortality (X). Participants were followed until 31 December 2010.

### Statistical analyses

The analyses were performed with SAS, version 9.2 (SAS Institute Inc. Cary, NC USA). All p-values were two-sided, and p-values<0.05 were considered statistically significant. Descriptive characteristics of the participants presented as percent (number) and corresponding UACR geometric mean (95% confidence interval, CI) are presented in table 1. The main causes of death and corresponding UACR levels are presented in table 2.

**Table 1:** Urine albumin creatinine status according to baseline characteristics of the study populations.

	Monica10		Inter99	
	% (n)	UACR, geometric mean (95% CI), mg/g	% (n)	UACR, geometric mean (95% CI), mg/g
Gender				
Male	50.2 (1332)	1.8 (1.7, 2.0)	49.2 (3183)	3.3 (3.2, 3.4)
Female	49.8 (1322)	2.6 (2.4, 2.8)	50.8 (3288)	4.0 (3.9, 4.1)
P-value$		<0.0001		<0.0001
Age, years				
≤45	27.4 (726)	1.7 (1.5, 1.8)	46.2 (2987)	3.5 (3.4, 3.6)
45–55	27.9 (741)	1.8 (1.6, 2.0)	39.0 (2524)	3.6 (3.5, 3.8)
≥55	44.7 (1187)	2.9 (2.6, 3.2)	14.8 (960)	4.2 (4.0, 4.5)
P-value$		<0.0001		<0.0001
Education				
No	25.5 (677)	2.7 (2.4, 3.0)	16.4 (1019)	3.8 (3.6, 3.9)
Yes	74.5 (1976)	2.0 (1.9, 2.2)	83.6 (5211)	3.6 (3.5, 3.7)
P-value$		<0.0001		0.12
Body mass index, kg/m^2^				
<18.5	1.0 (26)	4.3 (2.4, 7.8)	1.0 (67)	5.2 (4.3, 6.2)
18.5–24.9	45.3 (1203)	2.2 (2.1, 2.4)	42.4 (2740)	3.6 (3.5, 3.7)
25–29.9	38.1 (1012)	2.0 (1.8, 2.2)	39.4 (2549)	3.5 (3.4, 3.6)
≥30	15.6 (413)	2.2 (1.9, 2.6)	17.2 (1112)	4.4 (4.1, 4.6)
P-value$		0.005		<0.0001
Physical activity				
Sedentary	21.2 (552)	2.5 (2.2, 2.8)	21.7 (1369)	3.9 (3.8, 4.1)
Light	56.8 (1481)	2.2 (2.1, 2.4)	61.6 (3897)	3.7 (3.6, 3.8)
Moderate/vigorous	22.0 (573)	1.7 (1.5, 1.9)	16.7 (1057)	3.2 (3.1, 3.3)
P-value$		<0.0001		<0.0001
Smoking habits, g/day				
Never smoker	26.3 (695)	1.9 (1.7, 2.1)	35.4 (2269)	3.6 (3.4, 3.7)
Former smoker	27.5 (729)	2.2 (2.0, 2.5)	25.4 (1627)	3.6 (3.5, 3.8)
Current smoker, <15	19.9 (528)	2.3 (2.1, 2.6)	14.2 (912)	3.8 (3.6, 4.0)
Current smoker, <25	20.7 (547)	2.2 (2.0, 2.5)	18.2 (1168)	3.7 (3.5, 3.8)
Current smoker, ≥25	5.6 (147)	2.3 (1.7, 3.1)	6.8 (433)	4.0 (3.6, 4.4)
P-value$		0.07		0.37
Alcohol, drinks/week				
0	13.6 (359)	3.1 (2.7, 3.6)	9.8 (606)	4.1 (3.8, 4.4)
≤7	41.9 (1102)	2.4 (2.2, 2.6)	45.0 (2775)	3.6 (3.5, 3.7)
≤14	21.8 (573)	2.1 (1.9, 2.4)	21.6 (1332)	3.4 (3.3, 3.6)
>14	22.7 (599)	1.5 (1.3, 1.7)	23.6 (1458)	3.7 (3.5, 3.8)
P-value$		<0.0001		0.0006

$ Kruskal Wallis test.

Abbreviations: CI, confidence interval; UACR, urine albumin creatinine ratio.

**Table 2:** Main causes of death during follow-up in the Monica10 and Inter99 studies and UACR.

Cause of death (number of events)	Number of events	UACR, mg/g, Geometric mean (95% CI)
**Neoplasms C00–D48**	367	3.1 (2.7, 3.6)
Malignant neoplasms, digestive organs C15–C26	105	2.6 (1.9, 3.5)
Malignant neoplasm of bronchus and lung C34	93	2.9 (2.1, 4.0)
Malignant neoplasm of breast C50	34	3.5 (2.0, 6.1)
Other diagnoses included: malignant neoplasms, lip, oral cavity and pharynx C00–C14 (13); malignant neoplasms, female genital organs C51–C58 (18); malignant neoplasms, male genital organs C60–C63 (14); malignant neoplasms, urinary organs C64–C68 (19); malignant neoplasms, eye, brain and central nervous system C69–C72 (13); malignant neoplasms of lymphoid, haematopoietic and related tissue C81–C96 (21)		
**Endocrine, nutritional and metabolic diseases E00–E90**	19	23.4 (6.7, 81.8)
Diabetes mellitus E10–E14	14	44.5 (9.6, 206.5)
**Mental and behavioural disorders F00–F99**	32	3.8 (2.5, 5.7)
Dementia F00–F03	14	3.7 (2.0, 7.0)
Mental and behavioural disorders due to use of alcohol F10	16	3.7 (1.9, 7.4)
**Diseases of the nervous system G00–G99**	25	3.8 (2.3, 6.4)
Diagnoses included: Alzheimer's disease G30 (9)		
**Diseases of the circulatory system I00–I99**	247	4.5 (3.7, 5.6)
Ischemic heart diseases I20–I25	101	5.0 (3.4, 71)
Cerebrovascular diseases I60–I69	68	5.9 (3.9, 9.1)
Other diagnoses included: hypertensive diseases I10–I15 (11); heart failure I50 (9); diseases of arteries, arterioles and capillaries I70–I79 (15)		
**Diseases of the respiratory system J00–J99**	77	5.0 (3.4, 7.4)
Respiratory infections J00–J22	11	2.6 (1.0, 7.3)
Chronic obstructive pulmonary disease J44	50	7.2 (4.5, 11.5)
**Diseases of the digestive system K00–K93**	44	3.7 (2.0, 6.8)
Diseases of liver K70–K77	23	5.0 (2.2, 11.5)
Other diagnoses included: diseases of esophagus, stomach and duodenum K20–K31 (8)		

Abbreviations: CI, confidence interval; UACR, urine albumin creatinine ratio.

Data from the Monica10 and Inter99 studies were pooled. Multivariate Cox regression analysis was used to determine the association of baseline UACR and cause-specific mortality. UACR levels were used as a continuous variable (table 3) and divided into quartiles where the lowest quartile was used as reference (tables 4, 5, and 6). Estimates are presented as hazard ratios, HRs (95% CI). We used a complete case analysis (only participants with complete information on all considered variables were included for each outcome).

**Table 3:** Hazard ratios and 95% confidence intervals for the associations between UACR and cause-specific mortality (individuals included = 8,472, person years at risk = 95,598).

Death caused by	Events	Model 1& HR (95% CI) per 10 mg/g higher UACR	Model 2¤ HR (95% CI) per 10 mg/g higher UACR	Model 3# HR (95% CI) per 10 mg/g higher UACR
All-cause	859	1.01 (1.01, 1.02) P<0.0001	1.01 (1.01, 1.014) P<0.0001	1.01 (1.00, 1.01) P = 0.0002 P_quadr_ = 0.003
Neoplasms	346	1.01 (1.00, 1.02) P = 0.286	1.00 (0.99, 1.01) P = 0.661	1.00 (0.99, 1.01) P = 0.890 P_quadr_ = 0.071
Endocrine, nutritional and metabolic diseases	18	1.03 (1.02, 1.03) P<0.0001	1.03 (1.01, 1.04) P<0.0001	1.02 (1.00, 1.03) P = 0.031 P_quadr_ = 0.015
Mental and behavioural disorders	26	0.92 (0.66, 1.28) P = 0.628	0.93 (0.69, 1.24) P = 0.609	0.92 (0.67, 1.26) P = 0.594 P_quadr_ = 0.100
Diseases of the nervous system	24	1.00 (0.94, 1.06) P = 0.999	1.00 (0.93, 1.06) P = 0.928	1.00 (0.94, 1.06) P = 0.957 P_quadr_ = 0.438
Diseases of the circulatory system	230	1.01 (1.01, 1.02) P<0.0001	1.01 (1.01, 1.02) P<0.0001	1.01 (1.00, 1.01) P = 0.021 P_quadr_ = 0.082
Diseases of the respiratory system	70	1.01 (1.00, 1.02) P = 0.154	1.01 (1.00, 1.03) P = 0.168	1.01 (0.99, 1.03) P = 0.251 P_quadr_ = 0.227
Diseases of the digestive system	42	1.02 (1.01, 1.03) P = 0.0005	1.02 (1.01, 1.03) P = 0.0002	1.02 (1.00, 1.03) P = 0.013 P_quadr_ = 0.269

Participants with missing values in one of the used variables were excluded.

& Adjusted for gender and study population.

¤ Further adjusted for education, physical activity, smoking habits, body mass index, and alcohol consumption.

# Further adjusted for systolic blood pressure, fasting blood glucose and triglycerides.

P_quadr_ is the p-value for the quadratic term.

Abbreviations: CI, confidence interval; HR, hazard ratio; UACR, urine albumin creatinine ratio.

**Table 4:** Hazard ratios and 95% confidence intervals for the associations between UACR in quartiles and cause-specific mortality (individuals included = 8,472, person years at risk = 95,598).

Death caused by	Events	Model 1& HR (95% CI)	Model 2¤ HR (95% CI)	Model 3# HR (95% CI)
All-cause	859			
1^st^ UACR quartile		1 (reference)	1 (reference)	1 (reference)
2^nd^ UACR quartile		0.95 (0.76, 1.20)	0.97 (0.77, 1.23)	1.00 (0.80, 1.26)
3^rd^ UACR quartile		1.25 (1.02, 1.53)	1.24 (1.01, 1.52)	1.26 (1.02, 1.54)
4^th^ UACR quartile		1.77 (1.49, 2.10)	1.69 (1.43, 2.01)	1.56 (1.31, 1.86)
P-value$		P_trend_<0.0001	P_trend_<0.0001	P_trend_<0.0001 P_quadr_ = 0.197
Neoplasms	346			
1^st^ UACR quartile		1 (reference)	1 (reference)	1 (reference)
2^nd^ UACR quartile		0.81 (0.57, 1.15)	0.82 (0.57, 1.17)	0.82 (0.58, 1.17)
3^rd^ UACR quartile		1.04 (0.76, 1.42)	1.03 (0.75, 1.41)	1.03 (0.75, 1.41)
4^th^ UACR quartile		1.22 (0.92, 1.62)	1.16 (0.87, 1.54)	1.11 (0.83, 1.49)
P-value$		P_trend_ = 0.082	P_trend_ = 0.175	P_trend_ = 0.300 P_quadr_ = 0.330
Endocrine, nutritional and metabolic diseases	18			
1^st^ UACR quartile		1 (reference)	1 (reference)	1 (reference)
2^nd^ UACR quartile		4.18 (0.67, 25.92)	3.74 (0.60, 23.26)	4.79 (0.76, 30.44)
3^rd^ UACR quartile		2.40 (0.33, 17.62)	2.10 (0.29, 15.50)	2.81 (0.37, 21.08)
4^th^ UACR quartile		10.71 (2.31, 49.79)	10.04 (2.16, 46.69)	6.98 (1.43, 34.19)
P-value$		P_trend_ = 0.002	P_trend_ = 0.002	P_trend_ = 0.019 P_quadr_ = 0.714
Mental and behavioural disorders	26			
1^st^ UACR quartile		1 (reference)	1 (reference)	1 (reference)
2^nd^ UACR quartile		0.42 (0.048, 3.68)	0.41 (0.046, 3.62)	0.36 (0.036, 3.60)
3^rd^ UACR quartile		3.65 (1.19, 11.14)	3.39 (1.10, 10.46)	3.37 (1.09, 10.40)
4^th^ UACR quartile		2.42 (0.79, 7.43)	2.40 (0.78, 7.41)	2.34 (0.75, 7.31)
P-value$		P_trend_ = 0.033	P_trend_ = 0.036	P_trend_ = 0.043 P_quadr_ = 0.436
Diseases of the nervous system	24			
1^st^ UACR quartile		1 (reference)	1 (reference)	1 (reference)
2^nd^ UACR quartile		0.77 (0.15, 3.93)	0.71 (0.14, 3.64)	0.70 (0.14, 3.58)
3^rd^ UACR quartile		2.85 (0.97, 8.40)	2.72 (0.91, 8.15)	2.61 (0.87, 7.82)
4^th^ UACR quartile		1.60 (0.53, 4.87)	1.43 (0.46, 4.39)	1.43 (0.46, 4.45)
P-value$		P_trend_ = 0.196	P_trend_ = 0.285	P_trend_ = 0.285 P_quadr_ = 0.379
Diseases of the circulatory system	230			
1^st^ UACR quartile		1 (reference)	1 (reference)	1 (reference)
2^nd^ UACR quartile		1.20 (0.77, 1.87)	1.25 (0.80, 1.95)	1.30 (0.83, 2.04)
3^rd^ UACR quartile		1.17 (0.76, 1.79)	1.18 (0.77, 1.81)	1.22 (0.80, 1.87)
4^th^ UACR quartile		2.38 (1.72, 3.29)	2.36 (1.71, 3.27)	2.03 (1.46, 2.83)
P-value$		P_trend_<0.0001	P_trend_<0.0001	P_trend_<0.0001 P_quadr_ = 0.326
Diseases of the respiratory system	70			
1^st^ UACR quartile		1 (reference)	1 (reference)	1 (reference)
2^nd^ UACR quartile		1.01 (0.44, 2.33)	1.07 (0.47, 2.48)	1.09 (0.47, 2.51)
3^rd^ UACR quartile		1.36 (0.66, 2.79)	1.44 (0.69, 2.98)	1.43 (0.69, 2.98)
4^th^ UACR quartile		2.02 (1.13, 3.61)	1.96 (1.09, 3.52)	1.91 (1.06, 3.45)
P-value$		P_trend_ = 0.014	P_trend_ = 0.018	P_trend_ = 0.026 P_quadr_ = 0.729
Diseases of the digestive system	42			
1^st^ UACR quartile		1 (reference)	1 (reference)	1 (reference)
2^nd^ UACR quartile		0.72 (0.23, 2.24)	0.74 (0.24, 2.33)	0.82 (0.26, 2.58)
3^rd^ UACR quartile		1.30 (0.54, 3.13)	1.26 (0.52, 3.04)	1.28 (0.53, 3.12)
4^th^ UACR quartile		1.78 (0.84, 3.81)	1.68 (0.79, 3.59)	1.32 (0.61, 2.85)
P-value$		P_trend_ = 0.097	P_trend_ = 0.138	P_trend_ = 0.399 P_quadr_ = 0.863

Participants with missing values in one of the used variables were excluded.

& Adjusted for gender and study population.

¤ Further adjusted for education, physical activity, smoking habits, body mass index, and alcohol consumption.

# Further adjusted for systolic blood pressure, fasting blood glucose and triglycerides.

$ p_trend_ is the p-value for a linear trend, p_quadr_ is the p-value for the quadratic term.

Abbreviations: CI, confidence interval; HR, hazard ratio; UACR, urine albumin creatinine ratio.

**Table 5:** Hazard ratios and 95% confidence intervals for the associations between UACR and cause-specific mortality in the Monica10 study (individuals included = 2,569, person years at risk = 42,412).

Death caused by	Events	HR (95% CI)&
All-cause	662	
1^st^ UACR quartile		1 (reference)
2^nd^ UACR quartile		0.88 (0.67, 1.17)
3^rd^ UACR quartile		1.28 (1.02, 1.62)
4^th^ UACR quartile		1.64 (1.36, 1.97)
P-value$		P_trend_<0.0001 P_quadr_ = 0.074
Neoplasms	232	
1^st^ UACR quartile		1 (reference)
2^nd^ UACR quartile		0.69 (0.43, 1.10)
3^rd^ UACR quartile		1.10 (0.76, 1.59)
4^th^ UACR quartile		1.11 (0.81, 1.53)
P-value$		P_trend_ = 0.383 P_quadr_ = 0.313
Endocrine, nutritional and metabolic diseases	14	
1^st^ UACR quartile		1 (reference)
2^nd^ UACR quartile		5.63 (0.70, 45.46)
3^rd^ UACR quartile		6.24 (0.75, 52.20)
4^th^ UACR quartile		5.03 (0.93, 27.09)
P-value$		P_trend_ = 0.061 P_quadr_ = 0.196
Mental and behavioural disorders	19	
1^st^ UACR quartile		1 (reference)
2^nd^ UACR quartile		0
3^rd^ UACR quartile		2.56 (0.74, 8.83)
4^th^ UACR quartile		2.45 (0.77, 7.82)
P-value$		P_trend_ = 0.061 P_quadr_ = 0.859
Diseases of the nervous system	20	
1^st^ UACR quartile		1 (reference)
2^nd^ UACR quartile		0.96 (0.18, 5.10)
3^rd^ UACR quartile		2.98 (0.90, 9.87)
4^th^ UACR quartile		1.52 (0.45, 5.15)
P-value$		P_trend_ = 0.299 P_quadr_ = 0.286
Diseases of the circulatory system	193	
1^st^ UACR quartile		1 (reference)
2^nd^ UACR quartile		1.27 (0.77, 2.10)
3^rd^ UACR quartile		1.03 (0.62,1.70)
4^th^ UACR quartile		2.31 (1.64, 3.25)
P-value$		P_trend_<0.0001 P_quadr_ = 0.081
Diseases of the respiratory system	63	
1^st^ UACR quartile		1 (reference)
2^nd^ UACR quartile		0.94 (0.37, 2.36)
3^rd^ UACR quartile		1.68 (0.82, 3.46)
4^th^ UACR quartile		1.81 (0.99, 3.33)
P-value$		P_trend_ = 0.036 P_quadr_ = 0.953
Diseases of the digestive system	35	
1^st^ UACR quartile		1 (reference)
2^nd^ UACR quartile		0.30 (0.038, 2.28)
3^rd^ UACR quartile		1.70 (0.68, 4.23)
4^th^ UACR quartile		1.40 (0.62, 3.14)
P-value$		P_trend_ = 0.256 P_quadr_ = 0.843

& Adjusted for gender, education, physical activity, smoking habits, body mass index, alcohol consumption, fasting plasma glucose, systolic blood pressure, serum creatinine and serum triglycerides.

Abbreviations: CI, confidence interval; HR, hazard ratio; UACR, urine albumin creatinine ratio.

$ p_trend_ is the p-value for a linear trend, p_quadr_ is the p-value for the quadratic term.

**Table 6:** Hazard ratios and 95% confidence intervals for the associations between urine albumin and urine creatinine and all-cause mortality in the Monica10 study (individuals included = 2,569, person years at risk = 42,412).

		Urine albumin	Urine creatinine
Death caused by	Events	HR (95% CI)	HR (95% CI)
All-cause	662		
1^st^ quartile		1 (reference)	1 (reference)
2^nd^ quartile		0.88 (0.69, 1.11)	0.92 (0.75, 1.11)
3^rd^ quartile		1.16 (0.93, 1.46)	0.67 (0.53, 0.84)
4^th^ quartile		1.32 (1.06, 1.63)	0.72 (0.56, 0.93)
P-value$		P_trend_ = 0.001 P_quadr_ = 0.139	P_trend_ = 0.0005 P_quadr_ = 0.510

$ p_trend_ is the p-value for a linear trend, p_quadr_ is the p-value for the quadratic term.

We used age as underlying time axis and delayed entry where participants enter the analysis at the baseline age, and they exit the analysis at their event or censoring age. During follow-up, 1 participant disappeared and 68 participants emigrated. Participants were followed and contributed with risk time in the analyses until date of death, date of emigration, date of their last registered activity or 31 December 2010, whichever came first. In model 1 (tables 3, 4), we adjusted for study group and gender. Model 2 was further adjusted for education, physical activity, smoking habits, BMI, and alcohol consumption. Model 3 was further adjusted for systolic blood pressure, fasting plasma glucose and triglycerides.

Provided for each Cox regression analysis is in tables 3, 4, 5, and 6 the p-value for the linear term, or linear trend (p_trend_). The linear term refers to UACR as a continuous variable, whereas UACR quartiles are used for linear trend. However, because of a trend towards a U-shape across quartiles rather than a linear one for some of the outcome categories, we also provided the p-value for the quadratic term (p_quadr_) as a test for non-linearity in the fully adjusted models (tables 3, 4, 5, 6). The quadratic term refers to UACR (continuous or quartiles) squared. There was no interaction between UACR and gender, study group, or time since baseline examination. The proportional hazards assumption was checked visually.

In additional analyses (table 5), we further adjusted for serum creatinine (available in the Monica10 study only). We also performed separate analyses of urine albumin and urine creatinine as exposures in Monica10 for all-cause mortality (table 6).

## Results

In both cohorts, a higher UACR was associated with female gender, higher age, physical inactivity, and alcohol abstinence (table 1). The geometric means (95% CI) of UACR were 2.17 (2.05, 2.29) and 3.66 (3.59, 3.73) mg/g in the Monica10 and the Inter99 studies, respectively. The lowest UACR quartile was <2.0, the second quartile was 2.0-<3.0, the third quartile was 3.0-<5.0 and the highest quartile was ≥5.0 mg/g.

Medians (interquartile range) of systolic blood pressure, triglycerides and fasting plasma glucose were 127 (115–141) mmHg, 1.2 (0.9, 1.7) mmol/l and 4.7 (4.4, 5.1) mmol/l in the Monica10 study and 130 (120–140) mmHg, 1.1 (0.8, 1.6) mmol/l and 5.4 (5.1, 5.8) mmol/l in the Inter99 study. In the Monica10 study, 74 persons had self-reported diabetes, whereas the number was 129 in the Inter99 study. The median follow-up time was 11.3 years (Monica10: 16.5 years, Inter99: 11.0 years), and a total of 920 persons died; 692 from the Monica10 study and 228 from the Inter99 study.

Neoplasms and diseases of the circulatory system accounted for approximately 40%, and one fourth of deaths, respectively (table 2). In one third were the deaths caused by respiratory disease. UACR was highest among persons dying from endocrine, nutritional and metabolic diseases and lowest among people dying from neoplasms and diseases of the digestive system (table 2).

In the analyses that used UACR as a continuous variable, we found statistically significant positive associations between UACR status and risk of all-cause mortality, death caused by diseases of the circulatory system, diseases of the digestive system, and endocrine, nutritional and metabolic diseases with hazard ratios of 1.01, 1.01, 1.02, and 1.02, respectively, per 10 mg/g higher UACR (table 3). These estimates remained statistically significant through multiple adjustments. For all-cause mortality and endocrine, nutritional and metabolic diseases, there were statistically significant deviations from linearity (p_quadr_<0.05).

When analysing UACR in quartiles, we found statistically significant positive associations between UACR status and risk of all-cause mortality, endocrine nutritional and metabolic diseases, mental and behavioural disorders, diseases of the circulatory system, and diseases of the respiratory system (table 4), and the hazard ratios for the fourth UACR quartile with the first quartile as reference were 1.56, 6.98, 2.34, 2.03, and 1.91, respectively. The possible U-shape between UACR status and all-cause mortality and endocrine, nutritional and metabolic disorders implied by the deviations from linearity (table 3) was much less pronounced when analysing UACR in quartiles, and p_quadr_ is >0.05 for all outcomes in table 4.

To further explore the possible U-shape, we separately analysed the associations of urine albumin and urine creatinine with all-cause mortality. Urine albumin tended to follow a similar U-shape as UACR; urine creatinine showed an approximately linear decreasing trend across quartiles. None of these associations, however, showed statistically significant deviations from linearity (table 6).

The analysis of the association between UACR and death from endocrine, nutritional and metabolic diseases was essentially unchanged, when we excluded participants who died from endocrine, nutritional and metabolic diseases other than diabetes mellitus (N = 5). We only had 1 case of diabetic nephropathy (according to ICD-10: E10.2, E11.2, E12.2, E13.2, E14.2) among the participants that died from endocrine, nutritional and metabolic diseases so we were not able to take this complication into account.

In additional analyses (table 5), we further adjusted for serum creatinine (Monica10 only). Although performed on a much smaller sample size, the associations between UACR and all-cause mortality and death caused by diseases of the circulatory system and the respiratory system remained statistically significant, but the associations with death caused by endocrine, nutritional and metabolic diseases did not.

## Discussion

We aimed to investigate whether other causes of death than cardiovascular disease and diabetes contribute to the well-established positive association between UACR and all-cause mortality. We found statistically significant positive associations between baseline UACR and death from all-cause mortality, endocrine nutritional and metabolic diseases, diseases of the circulatory system, and possibly mental and behavioural disorders, and diseases of the respiratory and digestive system. The latter three were however only statistically significant in either the analysis with UACR as a continuous variable or in the analysis with UACR quartiles and should be interpreted with caution.

On death caused by circulatory and endocrine, nutritional and metabolic diseases, the observed positive and linear relationships with UACR levels are in line with previous studies [3], [23]. Likewise, our results of UACR and all-cause mortality are in line with previous studies. This association has been extensively studied in diabetics in particular –both micro- and normo-albuminurics. A collaborative meta-analysis of prospective general population studies found ACR 1.1 mg/mmol (10 mg/g) or more to be an independent predictor of mortality risk in the general population [3]. Our study is an extension of the previous studies since it also examines other specific causes of death than cardiovascular disease.

Regarding death caused by respiratory disease, our results are in line with Bulcun et al who found that UACR was significantly higher in patients with COPD than in controls [11]. Similarly, Casanova et al found microalbuminuria to be common in COPD patients and associated with hypoxemia independent of other cardiovascular risk factors [24]. The progressive airway limitation and destruction of pulmonary capillaries in COPD lead to the characteristic ventilation/perfusion abnormality which in turn causes hypoxemia. Hypoxia is thought to cause endothelial dysfunction which is closely related to albuminuria [11].

Albuminuria is considered a marker of small vessel disease and is associated with risk of hypertension, obesity and glucose levels [12], [25]. Cognitive decline is frequently attributed to microvascular disease in the brain, and the mentioned risk factors have been shown to predict dementia later in life [12]. The positive association between albuminuria and mental and behavioral disorders that mostly consists of dementia may therefore reflect the cumulative vascular damage over years related to hypertension, abnormal glucose metabolism, and other risk factor [12].

Regarding the possible U-shape of the association between UACR status and all-cause and endocrine, nutritional and metabolic disease mortality, it is somewhat in line with a large study by Kovesdy et al who reported a similar U-shape in patients with advanced CKD of the associations between UACR and all-cause mortality and progressive CKD [4]. They found that very low levels of UACR were associated with a higher risk in this subgroup –maybe reflecting an inability to adapt to lower renal perfusion pressures in CKD– and that the optimal range in this group was 10–19 mg/g. However, this explanation does not suffice in explaining why a similar U-shape is seen in our general population study. The U-shape may reflect the higher mortality among persons underweight and patients with other comorbidities.

Whether there is a safe threshold of albuminuria is still under debate because even urine albumin in the upper normal range bears a significant risk: albuminuria well below what is usually defined as microalbuminuria is a strong predictor of cardiovascular morbidity and mortality [14] and any degree of measurable albuminuria bears significant cardiovascular risk [9]. Likewise, a study found that a baseline urinary ACR ≥5 mg/g, a level not traditionally considered clinically significant, is independently associated with faster decline in cognitive function [26]. Thus, some authors suggest that UACR is used as a continuous variable rather than the traditionally classification of micro- and macroalbuminuria.

The strengths of our study include the longitudinal population-based design; the large general population sample used; the uniformity of the methods of UACR measurements in the merged studies; a long-term follow-up and the use of standardised registry-based diagnoses with almost no individuals lost to follow-up; and the available information on potential confounders.

The limitations of our study include the non-specific nature of the main causes of death; the interventional design in the Inter99 study; and the use of a single spot urine sample to assess UACR, which is less accurate than 24-hour urine collections [27], [28]. Both some acute (such as infections of the urinary tract) and chronic conditions may affect UACR [29] and serial measurements may have provided more accurate assessment of risk. Urine albumin levels are highly variable from day to day on a personal level and may benefit from repeated assessments to reduce the misclassification of albuminuria [30]. However, the misclassification is likely to be random and would tend to attenuate any true effect. Also, as the mortality in this general population sample is low, it gives a low number of events in some of the major causes of death, especially among the non-cardiovascular and non-neoplasm groups. Thus, the power for statistical analysis in some categories is low. Since participants were told about the results of their urine albumin creatinine measurements, participants with micro-albuminuria may be more likely to have undergone more detailed medical examinations, and, as a result, various asymptomatic diseases might have been detected earlier than otherwise. The chance of some diseases (including cognitive disorders and liver diseases) being diagnosed as the underlying cause of death might have been increased by such examinations. The relatively young ages and short follow-up times in the Inter99 study compared with the Monica10 study may have affected our results. Also, the UACR levels were different in the two cohorts which may be due to a general increase in prevalence of diabetes mellitus, impaired glucose tolerance and obesity that are all associated with a higher UACR [31]. However, the estimates were similar when the cohorts were analysed separately. The combined analyses were adjusted for study group, and there was no interaction between UACR and study group indicating that differences between the studies did not seriously affect our results.

Since albuminuria is quickly and non-invasively evaluated by a urine sample; is largely preventable; and has shown to be a consistent predictor of mortality, it is an important and interesting therapeutic target. There are several ways to prevent albuminuria or to prevent progression. The main therapy is a renin-angiotensin-aldosterone system blockade, which reduces proteinuria [32]. In normoalbuminuric patients, ACE inhibitors reduce the risk of developing microalbuminuria [33]. Likewise, angiotensin receptor blockers are believed to be able to prevent development of microalbuminuria. The Roadmap study, which is a multicenter phase 3 study designed to examine the effect of an angiotensin receptor blocker on prevention of microalbuminuria, found that treatment with olmesartan delayed the onset of microalbuminuria [34]. However, a higher risk of fatal cardiovascular events in the treatment group was a concern [35]. Also the vitamin D analog, paricalcitol, has shown to result in a decrease of albuminuria but a recent meta-analysis adviced caution in the use of any active vitamin D analogue in patients with CKD because of the potential risk of aggravating vascular calcification [36].

We found statistically significant positive associations between baseline UACR and death from all-cause mortality, endocrine nutritional and metabolic diseases, and diseases of the circulatory system and possibly mental and behavioural disorders, and diseases of the respiratory and digestive system. Also, we saw a tendency toward a U-shape in the association between UACR status and all-cause mortality and death from endocrine, nutritional and metabolic diseases. More studies are needed to further explore these associations.
